# Tapetal Expression of *BnaC.MAGL8.a* Causes Male Sterility in *Arabidopsis*

**DOI:** 10.3389/fpls.2019.00763

**Published:** 2019-06-12

**Authors:** Jie Gao, Qun Li, Nan Wang, Baolong Tao, Jing Wen, Bin Yi, Chaozhi Ma, Jinxing Tu, Tingdong Fu, Qiang Li, Jitao Zou, Jinxiong Shen

**Affiliations:** ^1^National Key Laboratory of Crop Genetic Improvement, National Engineering Research Center for Rapeseed, College of Plant Science and Technology, Huazhong Agricultural University, Wuhan, China; ^2^National Key Laboratory of Crop Genetic Improvement, College of Plant Science and Technology, Huazhong Agricultural University, Wuhan, China; ^3^National Research Council Canada, Saskatoon, SK, Canada

**Keywords:** *Arabidopsis thaliana*, *Brassica napus*, MAGL, male sterility, pollen wall, tapetum, *AMS*, jasmonates

## Abstract

Monoacylglycerol lipase (MAGL) hydrolyzes monoacylglycerol, producing free fatty acid and glycerol. Although this enzyme has been shown to play important roles in mammal, its potential function in plants remains poorly understood. In a survey of the *MAGL* genes in *Brassica napus*, we found tapetal expression of *BnaC.MAGL8.a*, a homolog of *AtMAGL8*, results in male sterility in *Arabidopsis thaliana*. Retarded tapetal PCD and defective pollen wall were observed in the transgenic plants. The tapetal cells became vacuolated at stage 9, and then degenerated at stage 11. Most microspores degenerated with the tapetal cells, and only few pollen grains with an irregular-shaped exine layer were produced in the transgenic plants. Transcriptome analysis identified 398 differentially expressed genes. Most of them are involved in pollen development and stress response. *ABORTED MICROSPORES* and its downstream pollen wall biosynthesis genes were down-regulated, but genes related with reactive oxygen species homeostasis and jasmonates signaling were up-regulated in the transgenic plants. These results suggest that expression of *BnaC.MAGL8.a* in tapetum invokes stress response and impairs pollen development. The apparent phenotypic similarity between *atgpat1* mutant and *BnA9::BnaC.MAGL8.a* transgenic plants lead us to propose a role for monoacylglycerol (MAG) in pollen development in *Arabidopsis*. Our study provides insights on not only the biological function of plant MAGL genes but also the role of MAG in pollen development.

## Introduction

In mammals, triacylglycerol (TAG) breakdown proceeds through sequential hydrolysis reactions catalyzed by adipose tissue triacylglycerol lipase, hormone sensitive lipase, and monoacylglycerol lipase (MAGL) (Labar et al., 2010). In addition to participate in fat mobilization, MAGLs also regulate the endocannabinoid signaling system by controlling the level of 2-arachidonoylglycerol, which is a key messenger of the endocannabinoid system (Labar et al., 2010). The first *MAGL* gene was cloned from mice (Karlsson et al., 1997), and then many *MAGL* identifications followed from various species (Karlsson et al., 2001; Heier et al., 2010). MAGLs belong to the alpha/beta hydrolase superfamily, containing the GXSXG motif and the catalytic triad of Ser, Asp, and His.

In *Arabidopsis*, a total of 16 putative *MAGL* genes were identified (Kim et al., 2016). MAG hydrolytic activity were measured with 11 recombinant proteins, among which, AtMAGL6 and AtMAGL8 exhibited the highest MAGL activities (Kim et al., 2016). The involvement of *AtMAGL8* in lipid mobilization is supported by its preferential expression in developing pollen and germinating seeds, the hydrolytic activity of AtMAGL8 toward the MAG containing eicosenoic acid (20:1), and its oil body associated localization (Kim et al., 2016). AtMAGL3 has two different enzymatic roles: lysophospholipase and monoacylglycerol acyltransferase (Gao et al., 2010; Vijayaraj et al., 2012). It is also named as lysoPL2 after its hydrolytic activity toward lysophosphatidylcholine and protein sequence similarity with lysoPL1 (Gao et al., 2010). AtMAGL3 was proposed to protect the plant from heavy metal ions by replacing oxidized membrane lipids with its protein partner acyl-CoA-binding protein 2 (ACBP2) (Gao et al., 2010).

The tapetum supports pollen development in an altruistic way, providing nutrients, enzymes, lipids and other materials. In the bicellular pollen stage, tapetal cells undergo programmed cell death (PCD) to convert their contents to building blocks for pollen development. Tapetal PCD is regulated by a number of transcription factors including *ABORTED MICROSPORES* (*AMS*) (Sorensen et al., 2003; Xu et al., 2010), *MALE STERILE 188/MYB103/MYB80* (*MS188/MYB103/MYB80*) (Higginson et al., 2003; Phan et al., 2012; Xiong et al., 2016) and *DYSFUNCTIONAL TAPETUM1* (*DYT1*) (Feng et al., 2012). Absence of these genes results in premature or retarded tapetal PCD and male sterility. *AMS* acts as a master regulator in pollen wall development, regulating 8 sporopollenin synthesis genes with *MS188* (Xiong et al., 2016; Wang et al., 2018). In addition to transcription factors, reactive oxygen species (ROS) also mediate tapetal PCD. It has been reported that the ROS levels peaked at stage 8 and 9 and then decreased at stage 11 in the rice anther (Hu et al., 2011). Similar ROS patterns were also observed in *Arabidopsis*, tobacco and tomato (Xie et al., 2014; Yu et al., 2017). *RESPIRATORY BURST OXIDASE HOMOLOG E* (*RBOHE*) encodes a NADPH oxidase producing ROS (Xie et al., 2014). Tapetal PCD was delayed in the *rbohe* mutant, and *RBOHE* overexpression resulted in premature tapetum degeneration (Xie et al., 2014).

Lipids are important for pollen development. The pollen wall comprises two layers, the exine and the intine (Ariizumi and Toriyama, 2011; Zhang et al., 2016). Lipid derived compounds are the major constitutes of the pollen coat and the exine (Ariizumi and Toriyama, 2011; Zhang et al., 2016). The pollen wall not only protects gamete from environmental stresses, but also plays an important role in pollen-stigma communication (Wang et al., 2017; Nasrallah, 2019). Defective pollen wall frequently results in male sterility. Many lipid related genes are important for pollen wall development, such as *MALE STERILITY2* (Aarts et al., 1997; Chen et al., 2011), *CYTOCHROME P450 FAMILY 703 SUBFAMILY A POLYPEPTIDE 2* (*CYP703A2*) (Morant et al., 2007), *CYP704B1* (Dobritsa et al., 2009), and *ACYL-COA SYNTHETASE 5* (*ACOS5*) (de Azevedo Souza et al., 2009). Moreover, loss of genes involved in glycerolipid synthesis also leads to male sterility. Glycerol-3-phosphate acyltransferases (GPATs) mediate the production of lysophosphatidic acids from glycerol-3-phosphate and acyl-CoA. *gpat1* and *gpat6* mutants in *Arabidopsis* (Zheng et al., 2003; Li et al., 2012) and *gpat3* mutant in rice (Men et al., 2017) are characterized with defective tapetum development and male sterility. Knockout of *GPAT9* also leads to male gametophytic lethality in *Arabidopsis* (Shockey et al., 2016).

In this work, we cloned *BnaC.MAGL8.a*, which encodes a MAG lipase, from an oilseed rape cultivar (Zhongshuang 11). *AtMAGL8* is a promising candidate gene related with lipid mobilization, and it is preferentially expressed in developing pollen and germinating seeds. In this study, we used the *BnA9* promoter and the cauliflower mosaic virus (CaMV) 35S promoter to drive *BnaC.MAGL8.a* expression in *Arabidopsis* to explore its potential biological function. Though the *35S::BnaC.MAGL8.a* transgenic plants exhibited similar phenotype as wild type, overexpression of *BnaC.MAGL8.a* in tapetum impaired pollen development. The tapetal cells became vacuolated at stage 10 and degenerated with microspores at later stages in the *BnA9::BnaC.MAGL8.a* transgenic plants. Transcriptome analysis uncovered 398 differentially expressed genes in comparison with the wild type, mainly involved in pollen development and stress response. Based on these results, we discussed the potential mechanism underlying male sterility in the transgenic plants.

## Materials and Methods

### Plant Materials and Growth Condition

Zhongshuang 11 (ZS 11), a *Brassica napus* cultivar, was grown in the experimental field of Huazhong Agricultural University, Wuhan, China. Wild-type *Arabidopsis thaliana* Columbia (ecotype Col-0) and *BnA9::BnaC.MAGL8.a* transgenic plants were grown in a greenhouse (8 h dark and 16 h light, 23°C).

### Protein Sequence Alignment

The deduced amino acid sequence of *AtMAGL8* and its four homologous genes were aligned using the ClustalW method in MEGA 7 (Kumar et al., 2016).

### Vector Construction and Plant Transformation

Total RNA was extracted from ZS 11 flower buds using RNeasy Plant Mini Kit (Qiagen) and reverse transcription was performed using RevertAid First Strand cDNA Synthesis Kit from Thermo Scientific. Then, the coding sequence of *BnaC.MAGL8.a* was amplified with BnaC.MAGL8.a_LP and BnaC.MAGL8.a_RP. The *BnA9* promoter was amplified from ZS 11 genomic DNA with BnA9_LP and BnA9_RP. The *NOS* terminator was amplified using NosT_LP and NosT_RP from the pCAMBIA2300 vector. Primers used in this study are listed in Supplementary Table S1. The produced fragments were assembled in the pCAMBIA2300 vector. Then, the vector was transferred into *Agrobacterium tumefaciens* strain GV3101 and transformed into *Arabidopsis* via the floral dip method (Zhang X. et al., 2006).

### Recombinant Protein Purification and *in vitro* Enzyme Assay

The coding sequences from *BnaC.MAGL8.a* and *AtMAGL8* were inserted into the pMAL-c5X vector from the MBP purification system kit (NEB). After confirmed by sequencing, the vectors were introduced into *E. coli* strain ER2523. Protein purification and western blot were performed according to the manufacturer’s instructions. After dialysis against 50 mM phosphate sodium solution (pH 8.0), the maltose binding protein (MBP):MAGL recombinant proteins were quantified using Bradford protein assay kit (Tiangen). The recombinant proteins were incubated with the substrates (Supplementary Table S2), MAGs with various fatty acids, including palmitic acid (16:0), palmitoleic acid (16:1), stearic acid (18:0), oleic acid (18:1), and linoleic acid (18:2), at *sn*-1 position and the MAG with 18:1 at *sn*-2 position in 100 μl reaction solution containing 50 mM sodium phosphate buffer (pH 8.0), 0.2% Triton X-100 (Kim et al., 2016). All substrates were bought from Sigma-Aldrich and were emulsified in 0.2% Triton X-100 solution before use. After 30 min incubation, the reaction was stopped by 90°C treatment for 5 min and the released free fatty acid was measured by NEFA kit (Wako).

### Alexander’s Staining of Pollen

Anthers were stained with Alexander solution (Alexander, 1969) and examined microscopically.

### Light and Electron Microscopy

The semi-thin sections were prepared and analyzed as previously described (Chen et al., 2009). Transmission electron microscopy (TEM) analysis and scanning electron microscopy (SEM) analysis were conducted as previously described (Yi et al., 2010).

### Transcriptome Analysis

Total RNA was extracted from young flower buds (stage 6–9) with RNAprep pure Plant Kit (Tiangen) from the wild type and transgenic lines. Three biological replicates for the wild type and *BnA9::BnaC.MAGL8.a* plants were included. Libraries were sequenced with HiSeq X Ten system. Hisat2 was used to map the reads to the *Arabidopsis* genome (Kim et al., 2015). During this process, *BnaC.MAGL8.a* coding sequence was added to the reference genome to get its expression value. Raw read counts were estimated by featureCount, and differentially expressed genes was detected via DESeq2 using *P*-value <0.01 as the threshold (Liao et al., 2014; Love et al., 2014). The data was deposited in GEO database (GSE124918). The Gene Ontology classification was conducted using GO annotation tool from TAIR^1^ (Berardini et al., 2004). The MAPMAN annotation was conducted according to the user’s manual (Thimm et al., 2004). DAVID was used for functional classification and KEGG pathway enrichment (Huang et al., 2009).

### Real-Time PCR

Total RNA was extracted from flower buds (stage 6–9) from the wild type and *BnA9::BnaC.MAGL8.a* plants. First-strand cDNA was synthesized using RevertAid First Strand cDNA Synthesis Kit (Thermo Fisher). Real-time PCR was performed using TOYOBO SYBR GREEN mix and Bio-Rad cycler. Experiments were conducted in triplicate. Primers were listed in Supplementary Table S1. *PP2AA3* (*PROTEIN PHOSPHATE 2A SUBUNIT 3*, *AT1G13320*) was used for standardization (Singer et al., 2016) and the relative expression levels were calculated with CFX Manager Software (Bio-Rad) according to the 2^−ΔΔCt^ method.

## Results

### Discovery of the *BnA9::BnaC.MAGL8.a* Male Sterile Plants

To explore the *MAGL* gene family in *Brassica napus*, 47 genes were identified from the Darmor-*bzh* genome database (Chalhoub et al., 2014) based on the protein sequence similarity with 16 AtMAGLs (Supplementary Figure S1). We have successfully cloned 23 genes. Then, these genes were inserted into an ectopic expression vector containing CaMV35S or *BnA9* promoter and introduced into *Arabidopsis*. Stable transgenic lines harboring 15 genes were obtained (Supplementary Table S3). The CaMV35S promoter is commonly used for ectopically expressing genes in transgenic plants, but it does not drive a sufficiently high level of expression in the tapetum. *BnA9* promoter drives gene expression in tapetum from stage 6 to stage 11 (Konagaya et al., 2008; Song et al., 2016). Lipid metabolism plays an important role in pollen development. To explore the affect of ectopic expression of *MAGL* genes in pollen development, *BnA9* promoter were used to drives tapetum specific gene expression. Among the transgenic lines, *BnA9::BnaC.MAGL8.a* and *BnA9::BnaA.MAGL10.a* exhibited male sterility (Supplementary Table S3 and Supplementary Figure S2). As we were unable to purify MBP:BnaA.MAGL10.a recombinant protein for enzyme assay, this study is focused on the *BnA9::BnaC.MAGL8.a* transgenic plants.

**FIGURE 1:**
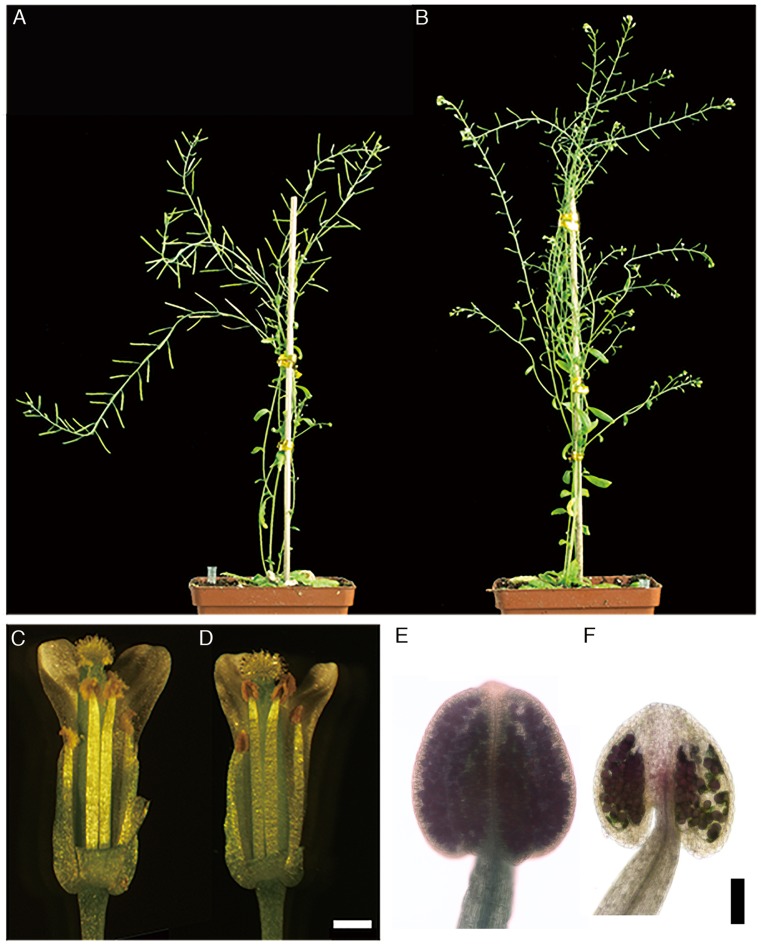
Phenotype of the *BnA9::BnaC.MAGL8.a* plants. **(A)** The wild type plant with long siliques. **(B)** The *BnA9::BnaC.MAGL8.a* plant with stunted siliques. Open flowers from the wild type **(C)** and the *BnA9::BnaC.MAGL8.a* plants **(D)**. The anthers of transgenic plants turn brown. Bar = 1 mm. Alexander’s staining of anthers from the wild type **(E)** and the *BnA9::BnaC.MAGL8.a* plants **(F)**. Bar = 100 μm.

In the *BnA9::BnaC.MAGL8.a* plants, the siliques were stunt and failed to set seeds (Figure 1A,B). Among the 12 *BnA9::BnaC.MAGL8.a* transgenic lines, 9 showed similar male-sterile phenotypes (Supplementary Table S4). The anthers from transgenic plants turned brown and no pollen grains were observed on the stigma or style (Figure 1D). In contrast, abundant pollen grains were observed in the wild type (Figure 1C). Alexander’s staining of anthers from the transgenic plants revealed that degenerated pollen grains adhered with round pollen grains in the locule (Figure 1E,F). Pollinating the *BnA9::BnaC.MAGL8.a* plants with wild type pollen grains led to normal siliques, suggesting the male sterility is caused by defective pollen grains (Supplementary Table S4).

### *BnaC.MAGL8.a* Is a Homologous Gene of *AtMAGL8* and Encodes a MAG Lipase

**FIGURE 2:**
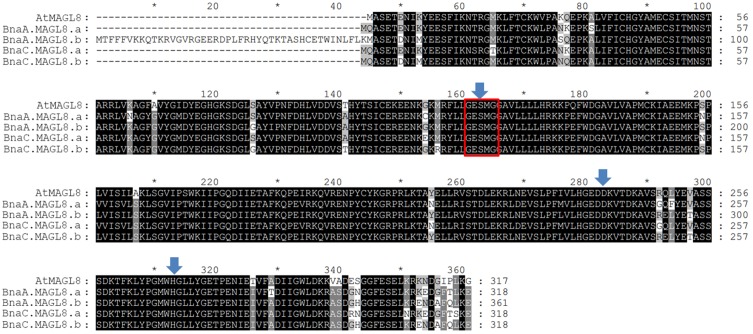
Alignment of deduced amino acid sequences of *AtMAGL8* and four homologous genes. These deduced amino acid sequences were aligned by ClustalW in MEGA 7. The identical and conserved amino acids residues are shaded in black and gray. The Gly-X-Ser-X-Gly (X = any residue) lipase motif is marked with red box and the catalytic triad (Ser, Asp, and His) is highlighted with blue arrows.

Based on phylogenetic analysis (Supplementary Figure S1), there are four homologous genes of *AtMAGL8* in *Brassica napus*, designated as *BnaC.MAGL8.a*, *BnaC.MAGL8.b*, *BnaA.MAGL8.a*, and *BnaA.MAGL8.b*. Alignment of the deduced protein sequences from these genes with *AtMAGL8* indicated that the GXSXG motif and the catalytic triad are conserved (Figure 2). The BnaC.MAGL8.a shows the highest identity (89%) with AtMAGL8. BnaC.MAGL8.b, BnaA.MAGL8.a, and BnaA.MAGL8.b exhibit 87.7, 87.7, and 80% identity with AtMAGL8, respectively.

**FIGURE 3:**
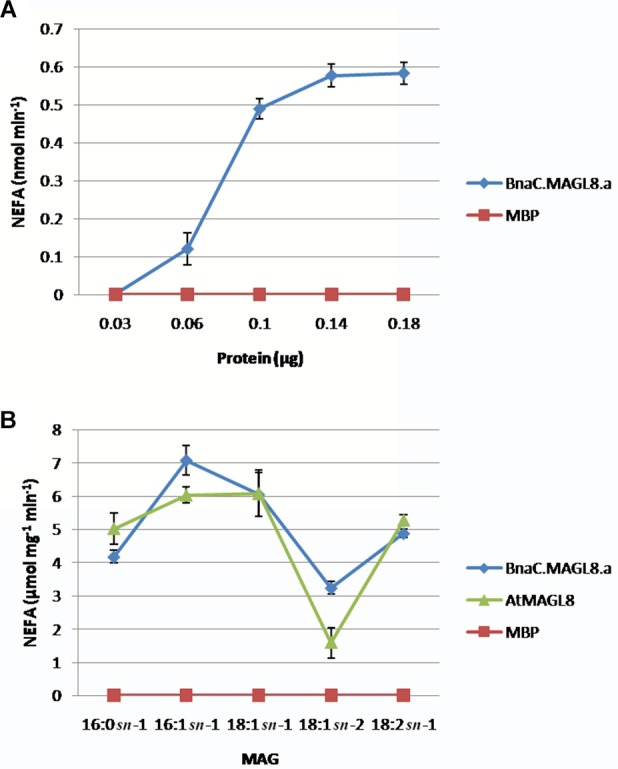
*In vitro* enzymatic assays of MBP:BnaC.MAGL8.a recombinant protein. **(A)** Protein-dependent MAGL assay were conducted with various amounts of recombinant protein (0.03–0.18 μg). The recombinant protein was incubated with 400 μM MAG containing 18:2 at the *sn*-1 position. **(B)** Substrate-dependent MAGL assay were conducted with 0.1 μg recombinant protein. The substrates were added to 400 μM in the reactions and released non-esterified fatty acid (NEFA) were measured using a NEFA assay kit. The values are means ± standard errors of three independent experiments.

*In vitro* enzyme assay indicated the MBP:BnaC.MAGL8.a recombinant protein possessed biochemical properties similar to MBP:AtMAGL8. The recombinant proteins were purified with amylose resin (Supplementary Figure S3). The production rate of non-esterified fatty acid increased with the MBP:BnaC.MAGL8.a recombinant protein content in the reaction and reached a maximum rate with 1.4 μg or more recombinant protein (Figure 3A), suggesting that BnaC.MAGL8.a and AtMAGL8 have a similar hydrolytic activity of MAG (Kim et al., 2016). We further investigated regio-specificity and substrate specificity of MBP:BnaC.MAGL8.a to different MAG species. Same as MBP:AtMAGL8, the MBP:BnaC.MAGL8.a recombinant protein preferred MAG with 18:1 at *sn*-1 position to *sn*-2 position. In addition, MBP:BnaC.MAGL8.a also showed a higher hydrolase activity with MAG containing unsaturated fatty acid (16:1) than saturated fatty acid (16:0) (Figure 3B). Hence, MBP:BnaC.MAGL8.a recombinant protein showed a similar regio-specificity and substrate specificity as MBP:AtMAGL8.

### Defective Pollen Development in the *BnA9::BnaC.MAGL8.a* Plants

**FIGURE 4:**
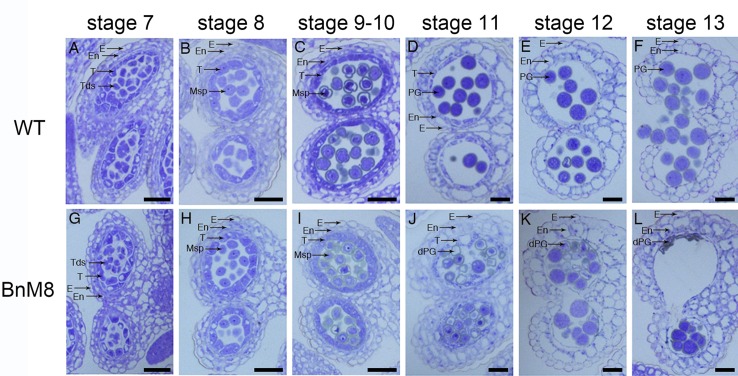
Transverse semi-thin sections of anthers from the wild type and *BnA9::BnaC.MAGL8.a* plants. **(A–F)** Anther from the wild type at stage 7 **(A)**, stage 8 **(B)**, stage 9 and 10 **(C)**, stage 11 **(D)**, stage 12 **(E)**, and stage 13 **(F)**; **(G–L)**: anther from the *BnA9::BnaC.MAGL8.a* plants at stage 7 **(G)**, stage 8 **(H)**, stage 9 and 10 **(I)**, stage 11 **(J)**, stage 12 **(K)**, and stage 13 **(L)**. BnM8, *BnA9::BnaC.MAGL8.a*; dPG, degenerated pollen grain; E, epidermis; En, endothecium; Msp, microspore; PG, pollen grain; T, tapetum; Tds, tetrads; WT, the wild type. Bars = 25 μm.

To unravel the mechanism of defective pollen formation, sections of anthers from the wild type and *BnA9::BnaC.MAGL8.a* plants were examined under light microscope. Normal microsporogenesis was observed in the transgenic plants (Figure 4A,B,G,H and Supplementary Figures S4A,E). In the *BnA9::BnaC.MAGL8.a* plants, microspores were released from the tetrads as the wild type at stage 8 (Figure 4H and Supplementary Figure S4E). At stage 9, the wild type tapetal cells initiated programmed cell death, evidenced by their reduced size (Figure 4C and Supplementary Figures S4B,C). However, in the transgenic plants, the tapetum became vacuolated and abundant small vacuoles were presented in the tapetal cells (Figure 4I and Supplementary Figure S4F). In addition, some of the microspores were degraded at this stage (Figure 4I and Supplementary Figure S4G). At stage 11, tapetum was degenerated in the wild type (Figure 4D,E and Supplementary Figure S4D). However, vacuolated tapetal cells and microspores filled the locules and large vacuoles were present in the tapetum of transgenic plants (Figure 4J and Supplementary Figure S4H). Some of the tapetal cells were degraded, leaving cell debris among the microspores. Most microspores were degraded, presented as empty exine ring in the section, and only a few still viable (Figure 4J). At stage 12, tapetal PCD were completed and pollen grains were freely distributed in the wild type (Figure 4E). In the *BnA9::BnaC.MAGL8.a* plants, the degenerated pollen grains were attached to normal ones and all of them appeared stuck to the locule wall (Figure 4K). At stage 13, only pollen debris and irregular pollen grains could be observed in the *BnA9::BnaC.MAGL8.a* plants (Figure 4L), when mature pollen grains were observed in the locules of wild type (Figure 4F).

**FIGURE 5:**
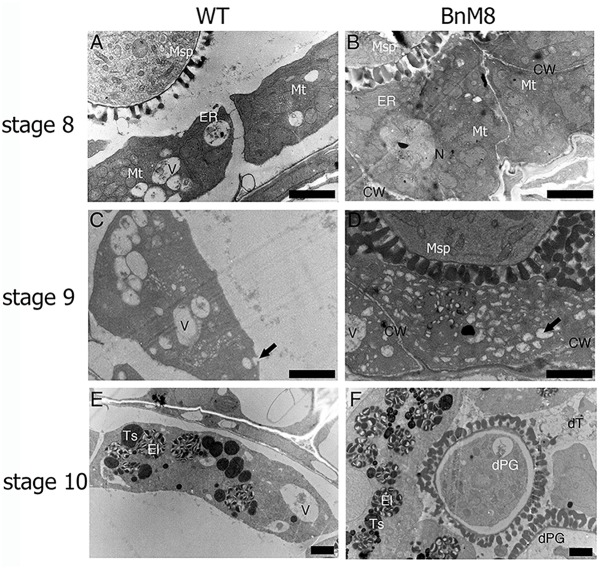
Transmission electron micrographs of the tapetum. **(A,C,E)** Tapetal cells from the wild type at stage 8 **(A)**, stage 9 **(C)**, and stage 10 **(E)**; **(B,D,F)**: tapetal cells from the *BnA9::BnaC.MAGL8.a* plants at stage 8 **(B)**, stage 9 **(D)**, and stage 10 **(F)**. BnM8, *BnA9::BnaC.MAGL8.a*; CW, cell wall; dPG, degenerated pollen grain; dT, degenerated tapetum; El, elaioplast; ER, endoplasmic reticulum; Ex, exine; In, intine; Mt, mitochondria; N, nucleus; Ts, tapetosome; V, vacuole; WT, the wild type. Arrow in **(C)** points to the tapetal cell releasing fibrillar materials. Arrow in **(D)** points to the small vesicle inside the tapetal cells. Bars = 2 μm.

Transmission electron microscope analysis was employed to examine the defective pollen development at subcellular level. In the wild type, layers of ER were presented beneath the plasma membrane facing the locule, indicating an active secretory function; in addition, the cell walls were degraded (Figure 5A). In the *BnA9::BnaC.MAGL8.a* plants, the outer tangential cell walls of tapetal cells were degraded and layers of ER were also observed (Figure 5B), but the cell wall between adjacent tapetal cells remained visible till stage 9 (Figure 5D). At stage 9, the wild type tapetum contained large vacuoles as well as many small vesicles, releasing fibrillar materials into the locule (Figure 5C). In the *BnA9::BnaC.MAGL8.a* plants, in addition to the large vacuoles, a great number of small vesicles were present in the tapetal cells, giving them a spongy appearance (Figure 5D). At stage 10, elaioplasts and tapetosomes were observed in the wild type and *BnA9::BnaC.MAGL8.a* plants (Figure 5E,F). Some of the tapetal cells were degenerated in the transgenic plants, leaving cell remnants in the locule (Figure 5F). Degenerated pollen grains were observed in the transgenic plants, featured with detached cytoplasm (Figure 5F).

**FIGURE 6:**
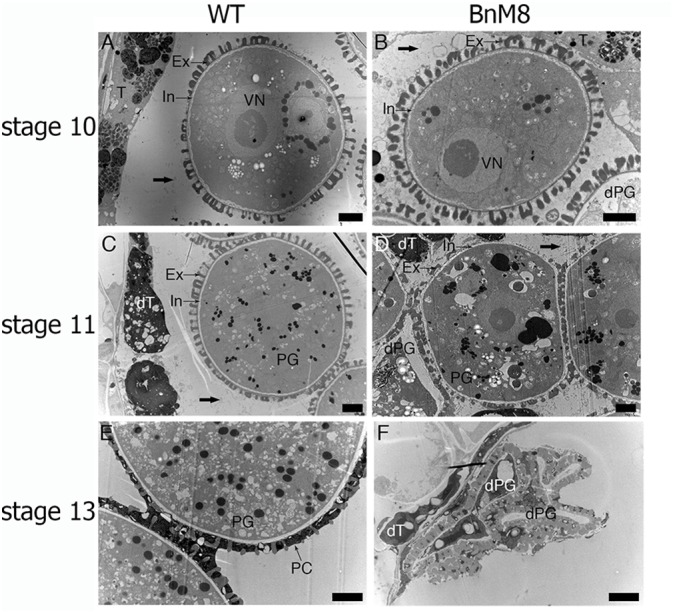
Transmission electron micrographs of microspores and pollen grains. **(A,C,E)** Microspores and pollen grains from the wild type in stage 10 **(A)**, stage 11 **(C)**, and stage 13 **(E)**; **(B,D,F)** microspores and pollen grains from the *BnA9::BnaC.MAGL8.a* plants in stage 10 **(B)**, stage 11 **(D)**, and stage 13 **(F)**. BnM8, *BnA9::BnaC.MAGL8.a*; dPG, degenerated pollen grain; dT, degenerated tapetum; Ex, exine; In, intine; PC, pollen coat; PG, pollen grain; VN, vegetative nucleus; WT, the wild type. Arrows in **(A,B,C,D)** point to the fibrillar materials in the locule. Bars = 2 μm.

Pollen wall formation was also affected in the *BnA9::BnaC.MAGL8.a* plants, especially the exine. Although some pollen grains from *BnA9::BnaC.MAGL8.a* appeared normal at stage 10, others were mostly degenerated in the locule (Figure 6B). At stage 11, the wild type pollen accumulated lipid droplets and starch granules (Figure 6C). On the other hand, large vacuoles presented in the pollen cytoplasm and the defect in exine became obvious in transgenic plants (Figure 6D). Furthermore, round pollen grains and degenerated pollen grains were attached to each other in the locule (Figure 6D). As the tapetal PCD was perturbed in the transgenic lines, limited pollen coat was uploaded onto the pollen wall (Figure 6F). Finally, after tapetum and pollen degradation, the collapsed pollen grains and tapetum remnants attached to the locule (Figure 6F). Notably, the locule fluid in the transgenic lines contained more fibrillar materials than the wild type at stage 10 and stage 11 (Figure 6B,D), which may came from degenerated tapetum and caused pollen grains aggregation.

**FIGURE 7:**
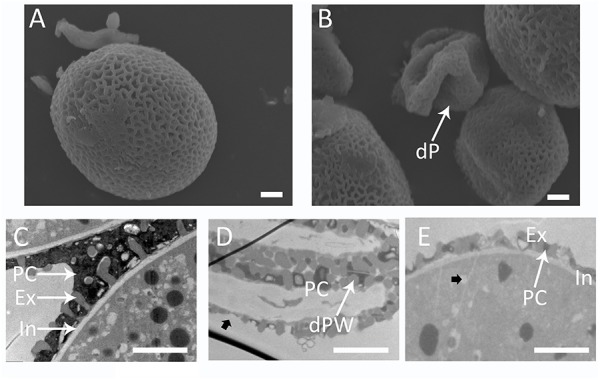
Defective pollen wall in the *BnA9::BnaC.MAGL8.a* plants. Scanning electron micrographs of pollen grains from the wild type **(A)** and the *BnA9::BnaC.MAGL8.a* plants **(B)**. Transmission electron micrographs emphasizing pollen wall of the wild type pollen **(C)**, and shrunken pollen grains **(D)** and round pollen grains **(E)** from the *BnA9::BnaC.MAGL8.a plants*. dP, degenerated pollen; dPW, degenerated pollen wall; Ex, exine; In, intine; PC, pollen coat. Arrow in **(D)** points to the thin exine layer in degenerated pollen. Arrow in **(E)** points to a storage organelle in the pollen cytoplasm. Bars = 2 μm.

Scanning electron microscopy analysis showed defective pollen wall in shrunken pollen grains. The exine was practically smooth and had small or no lacunae (Figure 7B). In addition, no intine or cytoplasm could be observed under the exine layer (Figure 7D). The round pollen grains were decorated with intine, exine and pollen coat (Figure 7E), and storage organelles were presented in the cytoplasm. However, the round pollen grains showed defective exine structure and limited pollen coat (Figure 7E).

### Transcriptome Analysis of the *BnA9::BnaC.MAGL8.a* Flower Buds

To assess the genes associated with male sterility in the *BnA9::BnaC.MAGL8.a* plants, transcriptome analysis was performed with young flower buds (stage 6–9) from the wild type and transgenic plants. In addition to *BnaC.MAGL8.a*, 397 differentially expressed genes (DEGs) were identified, composing 157 up-regulated genes and 240 down-regulated genes (Supplementary Table S5). The result of transcriptome analysis was validated with real-time PCR conducted on 6 selected genes (Supplementary Figure S5). Then, the DEGs were annotated with GO and MAPMAN (Berardini et al., 2004; Thimm et al., 2004). GO enrichment analysis showed that up-regulated genes were mainly enriched in response to stress, while down-regulated genes were mainly involved in developmental processes (Table 1). KEGG pathway enrichment analysis indicated that the DEGs were mainly localized in four pathways: biosynthesis of secondary metabolites, glucosinolate biosynthesis, phenylpropanoid biosynthesis and 2-oxocarboxylic acid metabolism (Table 2).

**Table 1:** Gene Ontology classification of DEGs.

GO term Biological process	Number of differentially expressed genes		
	Total	Down-regulated	Up-regulated
Response to stress	86	41	45
Response to abiotic or biotic stimulus	75	35	40
Developmental processes	58	44	14
Cell organization and biogenesis	53	34	19
Protein metabolism	47	33	14
Transcription, DNA-dependent	44	31	13
Transport	40	25	15
Signal transduction	30	23	7
DNA or RNA metabolism	6	5	1
Electron transport or energy pathways	6	2	4
Other cellular processes	202	121	81
Other metabolic processes	192	114	78
Other biological processes	66	34	32
Unknown biological processes	61	40	21

**Table 2:** Enriched KEGG pathways for differentially expressed genes.

	KEGG_ID	Pathway name	Count	Total	%	Fold enrichment	*P*-value
Up regulated	ath01110	Biosynthesis of secondary metabolites	20	38	12.99	2.34	0.0001
	ath00966	Glucosinolate biosynthesis	3	38	1.95	31.47	0.0037
	ath00940	Phenylpropanoid biosynthesis	6	38	3.90	4.81	0.0067
	ath01210	2-Oxocarboxylic acid metabolism	4	38	2.60	6.81	0.0189
Down regulated	ath00940	Phenylpropanoid biosynthesis	6	38	2.50	4.81	0.0067

Notably, six up-regulated genes and six down-regulated genes are involved in the phenylpropanoid biosynthesis pathway (Supplementary Table S6 and Supplementary Figure S6). The up-regulated genes are *PEROXIDASE 15* (*PRX15*), *PRX22*, *PRX49*, *PRX52*, *PRX72*, and *AT5G20940* (glycosyl hydrolase family protein). *PRX49*, *PRX52*, and *PRX72* participate in lignin synthesis progress and their up-regulated expression suggested an enhancement in lignin formation (Herrero et al., 2013a,b; Fernandez-Perez et al., 2015a,b). *CYP98A8* and *TAPETUM-SPECIFIC METHYLTRANSFERASE 1* (*TSM1*) are involved in spermidine synthesis, which is an important constituent for the pollen wall. The down-regulation of *CYP98A8* and *TSM1* in transgenic plants indicated that the phenylpropanoid polyamine conjugates synthesis was repressed in the transgenic plants.

Phenylpropanoid biosynthesis and glucosinolate biosynthesis are regulated by jasmonates signals as wound responses (Gaquerel et al., 2014; Wasternack and Strnad, 2019). *VEGETATIVE STORAGE PROTEIN1* (*VSP1*), a jasmonates response gene, was induced in transgenic plants. We compared our DEGs list with jasmonates regulated genes (JRGs) and 12-oxo-phytodienoic acid (OPDA) regulated genes (ORGs) (Taki et al., 2005). In total, 26 JRGs and five ORGs were differentially expressed in the transgenic plants (Supplementary Table S7). Of the 26 JRGs, 23 were also induced in wound response (Taki et al., 2005). Moreover, 11 of them were up-regulated in jasmonates treated stage 12 flowers (Supplementary Table S7) (Mandaokar et al., 2006). Based on this result, we propose that jasmonates, instead of OPDA, induced stress response in the transgenic plants.

Reactive oxygen species homeostasis genes showed altered expression in the transgenic plants. 19 ROS homeostasis genes were differentially expressed in the transgenic plants, and only three of them were down-regulated (Supplementary Table S8). Six ROS scavengers were up-regulated, including *FERRITIN 1* (*FER1*), *FER3*, *FER4*, *GLUTAREDOXIN 5*, *THIOREDOXIN-DEPENDENT PEROXIDASE 2* (*TPX2*), and *FE SUPEROXIDE DISMUTASE 1* (Mittler et al., 2004). *FER1*, *FER3*, and *FER4* function as a link between iron homeostasis and oxidative stress (Briat et al., 2010). As free iron induces harmful free radical production via the Fenton reaction, ferritins reduce ROS production by binding free irons in the cytoplasm (Ravet et al., 2009). *GLUTATHIONE S-TRANSFERASE PHI 9* and *GLUTATHIONE S-TRANSFERASE TAU 20* also reduce ROS levels (Noctor et al., 2011). The up-regulation of ROS scavenger genes suggested an unbalanced ROS homeostasis and activated ROS response in the transgenic plants.

Histology analysis indicated that pollen wall development was impaired in the transgenic plants. Consistent with this, 21 down regulated genes are among the 98 genes essential for pollen wall development (Table 3; Xu et al., 2014). As a master regulator in pollen wall formation, AMS directly associate with the promoters of *QUARTET3* (*QTR3*), *3-KETOACYL-COA SYNTHASE7* (*KCS7*), *KCS21*, *CYP98A8*, *CYP704B1*, and *EXTRACELLULAR LIPASE 5* (*EXL5*) (Xu et al., 2014). In the *BnA9::BnaC.MAGL8.a* plants, *AMS* and its six down-stream genes were down-regulated (Table 3). Moreover, *AMS*, working with *MS188*, regulates eight sporopollenin biosynthesis genes during pollen wall formation, including *ACOS5*, *CYP703A2*, *CYP704B1*, *MS2*, *POLYKETIDE SYNTHASE A* (*PKSA*), *PKSB*, *TETRAKETIDE α-PYRONE REDUCTASE1* (*TKPR1*), and *TKPR2* (Wang et al., 2018). According to transcriptome analysis result, only *CYP704B1* was significantly down-regulated, but all of them tend to express at lower levels in the *BnA9::BnaC.MAGL8.a* transgenic plants (Supplementary Table S9). Real-time PCR indicated that all of the eight genes were differentially expressed in the wild type and transgenic plants. *CYP703A2*, *PKSA*, and *TKPR1* were up-regulated and *CYP704B1*, *ACOS5*, *MS2*, *PKSB*, and *TKPR2* were down-regulated in the transgenic plants. Although the real-time PCR result and transcriptome analysis result are not perfectly matched, they suggested that *CYP704B1* and several sporopollenin biosynthesis genes were down regulated in transgenic plants and may lead to the irregular exine layer.

**Table 3:** Down-regulated gene involved in pollen wall formation.

TAIR ID^a^	Name	Description	Log_2_foldchange	*P*-value
AT2G16910	*AMS*	bHLH transcription factor	−0.76	0.0004
AT3G26125	*CYP86C2*	Cytochrome P450 86C2	−1.58	0.0001
AT1G69500	*CYP704B1^b^*	Cytochrome P450 704B1	−1.54	0.0013
AT1G28430	*CYP705A24*	Cytochrome P450 705A24	−1.53	0.0012
AT1G75920	*EXL5^b^*	Family II extracellular lipase 5	−1.44	0.0002
AT5G53190	*SWEET3*	Nodulin MtN3 family protein	−1.36	0.0049
AT1G33430	*KNS4/UPEX1*	Galactosyltransferase family protein	−1.28	<0.0001
AT4G14815		Lipid transfer protein family protein	−1.26	<0.0001
AT1G22015	*DD46*	Galactosyltransferase	−1.04	0.0042
AT4G29250		Transferase family protein	−1.00	0.0001
AT5G07530	*GRP17*	Glycine-rich protein 17	−0.99	0.0012
AT5G16960		NADP-dependent oxidoreductase, putative	−0.92	0.0001
AT1G26710		Transmembrane protein	−0.90	0.0038
AT1G71160	*KCS7^b^*	β-Ketoacyl-CoA synthase 7	−0.90	0.0005
AT4G20050	*QRT3^b^*	Polygalacturonase	−0.87	0.0003
AT1G74540	*CYP98A8^b^*	Cytochrome P450 98A8	−0.74	0.0066
AT1G75940	*ATA27*	Hydrolase, hydrolyzing O-glycosyl compounds	−0.69	0.0020
AT1G61110	*NAC25*	NAC transcription factor related with GA signal	−0.68	0.0037
AT5G49070	*KCS21^b^*	β-Ketoacyl-CoA synthase 21	−0.57	0.0049
AT5G48210		Unknown protein	−0.57	0.0077
AT1G67990	*TSM1*	Tapetum-specific O-methyltransferase	−0.56	0.0096

*^a^Locus identifier from TAIR (www.arabidopsis.org). ^b^Genes regulated by AMS (Xu et al., 2014).*

## Discussion

Since the discovery of mice *MAGL* in 1960, many other species have been proved to have multiple *MAGL* genes (Karlsson et al., 1997; Labar et al., 2010; Li-Beisson et al., 2013; Kim et al., 2016). MAGLs not only participate in fat mobilization, but also play important roles in endocannabinoid signaling in mammals (Labar et al., 2010). As a widespread gene family, the *MAGL* genes may be involved with many biological processes in plants. In *Arabidopsis*, 16 *MAGL* genes were characterized biochemically and molecularly (Kim et al., 2016). However, besides *AtMAGL3*, the potential functions of the *MAGL* genes have not been investigated in mutants or transgenic lines (Gao et al., 2010; Vijayaraj et al., 2012; Vanholme et al., 2013). In this study, we showed that expressing *BnaC.MAGL8.a* in tapetum affected tapetal PCD and microspore development, leading to male sterility in *Arabidopsis*.

### Retarded Tapetal PCD Was Correlated With Altered Expression of *AMS* and ROS Homeostasis Genes in the *BnA9::BnaC.MAGL8.a* Plants

An obvious difference between the *BnA9::BnaC.MAGL8.a* plants and the wild type is the vacuolated tapetum. The tapetum of transgenic plants contained many small vacuoles at stage 9 and 10. Meanwhile, the cytoplasm of several microspores receded from their pollen wall. The tapetum vacuolation became more severe at stage 11. Tapetal PCD is controlled by *DYT1*, *AMS*, *MALE STERILE1* and other transcription factors (Zhang W. et al., 2006; Ito et al., 2007; Yang et al., 2007; Xu et al., 2010). Their mutation caused defective tapetal PCD and tapetum vacuolation. The reduced *AMS* expression in the *BnA9::BnaC.MAGL8.a* plants was confirmed by transcriptome analysis and real-time PCR. *DYT1*, *AMS*, and *MS188* constitute a regulation pathway tuning tapetal PCD (Wang et al., 2018). The real-time PCR result indicated that *MS188* was significantly down-regulated in the transgenic plants and *DYT1* showed similar expression level in the wild type and transgenic plants. *BASIC HELIX LOOP HELIX PROTEIN 10* (*bHLH010*), *bHLH089*, and *bHLH091* are needed for *DYT1* to regulate anther development (Zhu et al., 2015). *bHLH010*, *bHLH089*, and *bHLH091* were significantly down-regulated in the transgenic plants, confirmed by the real-time PCR analysis. Moreover, down-regulated *bHLH091* expression was also supported by transcriptome analysis result (Supplementary Table S9). The down-regulation of *bHLH010*, *bHLH089* and *bHLH091* may repressed *AMS* expression.

In addition to transcription factors, tapetal PCD is also related with the ROS levels (Hu et al., 2011; Xie et al., 2014; Yu et al., 2017). The similar developmental changes in ROS profiles were found in *Arabidopsis*, tobacco and tomato (Xie et al., 2014; Yu et al., 2017). In addition, the manipulation of *RESPIRATORY-BURST OXIDASE HOMOLOG E* (*RBOHE*) changed anther ROS levels and resulted in defective tapetal PCD (Xie et al., 2014). BnaC.MAGL8.a may produce excessive free fatty acid (FFA) in the tapetum, same as AtMAGL3 hydrolyzing membrane lipids (Gao et al., 2010). There are two possible ways for FFA to affect ROS homeostasis, in the form of FFA or lipid derived oxylipins (Bonaventure, 2014). FFA may interact with respiratory chain, change the mitochondrial membrane fluidly and oxidizes glutathione inside the mitochondria (Schonfeld and Wojtczak, 2008). Under stress conditions, FFA could be transformed into oxylipins, including OPDA and jasmonates (Wasternack, 2007; Wasternack and Strnad, 2019). Based on our transcriptome data, large numbers of jasmonates-regulated genes were differentially expressed in the transgenic plants, suggesting that it is jasmonates, not OPDA, caused altered genes expression. *TPX2* and *GLUTATHIONE S-TRANSFERASE PHI 9* are regulated by jasmonates and function as ROS scavengers. The up-regulated ROS homeostasis genes in transgenic plants supported a ROS levels change in the transgenic plants. Notably, *RBOHE* is regulated by *AMS* and *MS188*. Hence, the regulator in tapetal PCD may initiate PCD process via regulating ROS levels (Xie et al., 2014). FFA, jasmonates and *AMS* regulated ROS levels in transgenic plants and the change in ROS level may also lead to a delayed tapetal PCD.

### Defective Pollen Wall Formation Was Correlated With Reduced Sporopollenin Synthesis Genes Expression in the *BnA9::BnaC.MAGL8.a* Plants

Many sporopollenin synthesis and pollen wall formation related genes were down-regulated in the transgenic plants (Table 3). The sporopollenin precursor is synthesized in the tapetum and then transported to the surface of microspores for exine wall formation. The sporopollenin is mainly composed of biopolymers derived from fatty acids and phenolic compounds (Ariizumi and Toriyama, 2011; Quilichini et al., 2015; Zhang et al., 2016). *CYP704B1* mediates long chain fatty acids hydroxylation (Dobritsa et al., 2009). *ACOS5*, *PKSA*, *PKSB*, and *TKPR1* are localized in the ER of tapetal cells and also in the locule (Grienenberger et al., 2010; Kim et al., 2010; Wang et al., 2018). It was proposed that these genes function as a metabolon for sporopollenin synthesis (Lallemand et al., 2013). Our real-time PCR result showed that *ACOS5, CYP704B1*, *MS2*, *TKPR2*, and *PKSB* were down-regulated in the transgenic plants (Supplementary Table S9), indicating a reduced sporopollenin synthesis. In phenylpropanoid biosynthesis pathway, *CYP98A8* and *TSM1* are involved in phenylpropanoid spermidine conjugates synthesis (Fellenberg et al., 2009) and the peroxidase genes, *PRX49*, *PRX52*, and *PRX72*, participate in lignin synthesis. The down-regulated *CYP98A8* and *TSM1* and up-regulated *PRX49*, *PRX52*, and *PRX72* in the transgenic plants may drive more phenylpropanoid compounds to lignin synthesis and reduced spermidine compounds production. The expression changes in sporopollenin related genes may resulted in irregular exine structure in the transgenic plants.

As the tapetal PCD was delayed in the transgenic lines, only limited pollen coat was loaded onto the pollen wall. In consistency with this, transcriptome analysis showed that some pollen coat related genes were down-regulated in the transgenic plants. GRP17 is the most abundant pollen coat protein in *Arabidopsis* and EXL5 is characterized as an extra cellular lipase in the pollen coat (Mayfield and Preuss, 2000; Mayfield et al., 2001). Down-regulation of *GRP17* and *EXL5* during early pollen stage may also affect pollen coat formation. *Tunicamycin induced 1* mutant shows altered pollen surface structure and the pollen grains are sticky (Iwata et al., 2012). Down-regulation of *TUNICAMYCIN INDUCED 1* may contribute to the defective pollen wall in transgenic plants (Supplementary Table S5).

In the *BnA9::BnaC.MAGL8.a* plants, the sticky pollen grains may caused by the defective pollen wall and the thick locule fluid. Mutation of sporopollenin synthesis genes results defective pollen grains. *cyp704b1* produces sticky pollen grains (Dobritsa et al., 2011). *cyp703a2* and *tkpr1* are characterized with thin-exine pollen wall (Dobritsa et al., 2011). The repressed sporopollenin synthesis genes expression correlated with thin and destructed exine layer in transgenic plants. As tapetum degenerated with microspores during stage 10 and stage 11, the locule fluid contained cell debris and cellular components, indicated as fibrillar materials in Figure 6. This change promoted pollen grains adhesion. As the microspores from *BnA9::BnaC.MAGL8.a* plants coalesced with each other in stage 11, when the pollen coat substrates was still enclosed in tapetum, the sticky pollen phenotype was more likely caused by defective pollen wall (Figure 6D). This notion was further supported by the fact that limited pollen coat was loaded on round or shrunken pollen grains (Figure 7).

### The Potential Role of MAG in Pollen Development

At stage 8, microspores are separated from the tapetum and obtain nutrients from the locule fluid. The exact composition of the locule fluid remains poorly understood. MAG, as a transportable metabolite, may serve as a locule fluid component. Arburscular mycorrhizal fungi and its host plant evolve a symbiosis relationship: the fungi transports mineral nutrients to the plant and receives MAGs and sugars as feedback (Jiang et al., 2017; Luginbuehl et al., 2017). *ram2* mutant in *Medicago truncatula* is defective in mycorrhizal fungal colonization and addition of C16 fatty acids complements the mutation (Wang et al., 2012). Further analysis indicated that *RAM2* encodes a GPAT synthesizing 2-monoacylglycerol, which likely exported to the fungi (Wang et al., 2012). Pollen development requires plenty of lipids (Zhang et al., 2016). As a high concentration of free fatty acid is toxic to plant cells, transporting MAG from tapetum could be a safe and efficient way to deliver lipid derived compounds to microspores. In the *atgpat1* mutant, the tapetum became hypertrophic and even occupied half of the locule (Zheng et al., 2003). Then, the microspores and tapetum degenerated, leaving collapsed pollen walls and several round pollen grains attached to the locule (Zheng et al., 2003). The similar phenotype of the *atgpat1* mutant and the *BnA9::BnaC.MAGL8.a* transgenic plants suggested a common mechanism for defect pollen development. We speculate that MAG could be the link, as the mutation of *AtGPAT1* and the *BnaC.MAGL8.a* overexpression may cause a MAG shortage. Further investigation is needed to prove the importance of MAG for microspore development.

## Author Contributions

JG, JZ, and JS contributed to the conception and design of the study. JG, QL, NW, and BT conducted the plant transformation and histology analysis. JG performed the enzyme assay and transcriptome analysis. JG wrote the first draft of the manuscript. BY, CM, JT, TF, and QL provided the valuable suggestions for this research. All authors contributed to the manuscript revision, read and approved the submitted version.

## Conflict of Interest Statement

The authors declare that the research was conducted in the absence of any commercial or financial relationships that could be construed as a potential conflict of interest.

## Footnotes

1 https://www.arabidopsis.org/tools/bulk/go/index.jsp
