# Supplementary material for: Tapetal Expression of BnaC.MAGL8.a Causes Male Sterility in Arabidopsis
Source: Front Plant Sci. 2019 Jun 12;10:763. doi: 10.3389/fpls.2019.00763 (PMC6582705; doi:10.3389/fpls.2019.00763)
Supplement: Supplementary file 1 [file Data_Sheet_1.PDF]

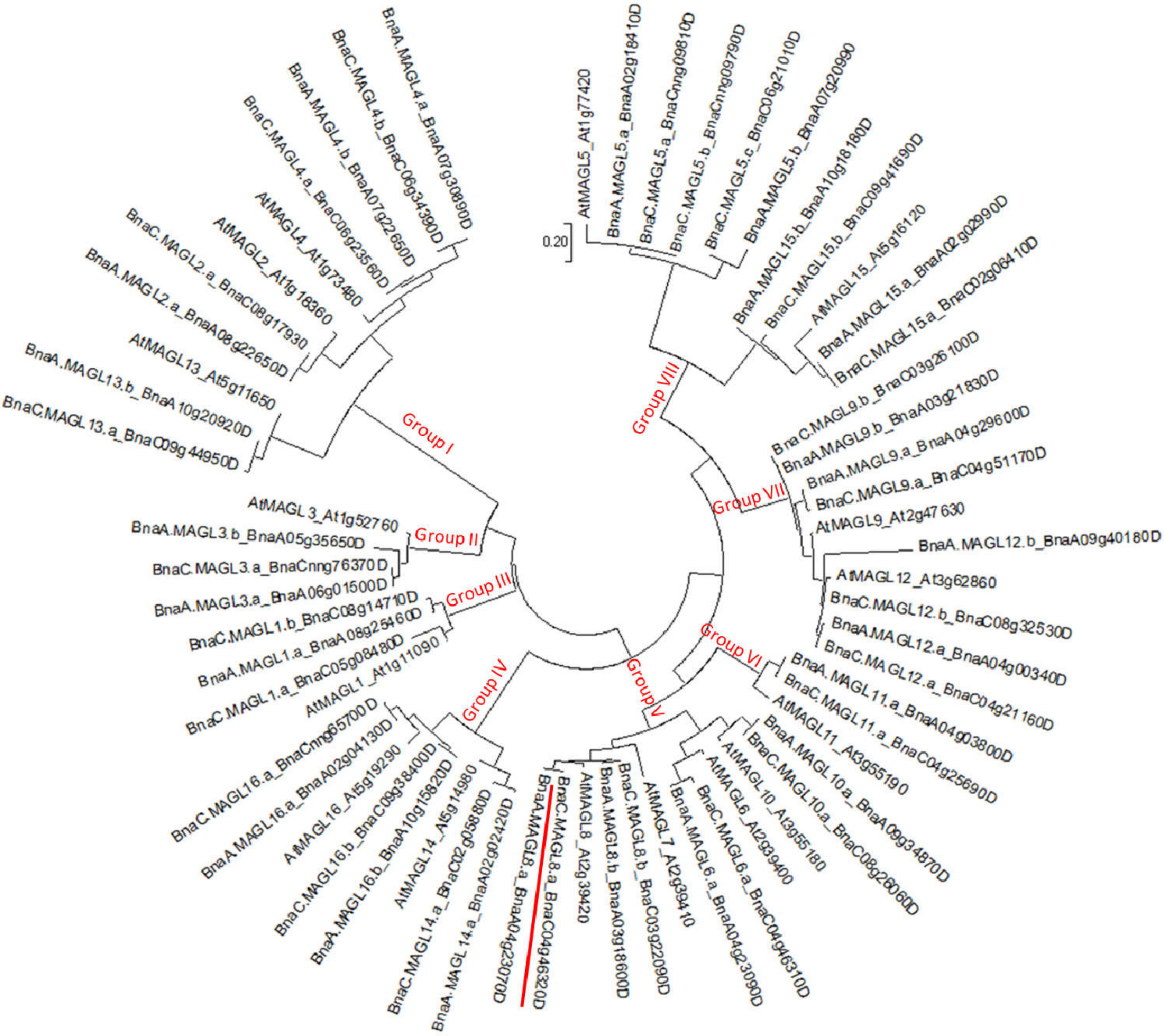

Figure S1. A phylogenetic tree of 47 Brassica napus putative MAGLs proteins and 16 Arabidopsis MAGL proteins. The phylogenetic tree was generated from deduced amino acid sequences of *MAGL* genes in *Brassica napus* and *Arabidopsis thaliana* using the Maximum Likelihood method based on the JTT matrix-based model in MEGA 7. The BnaC.MAGL8.a is highlighted with red bottom line.

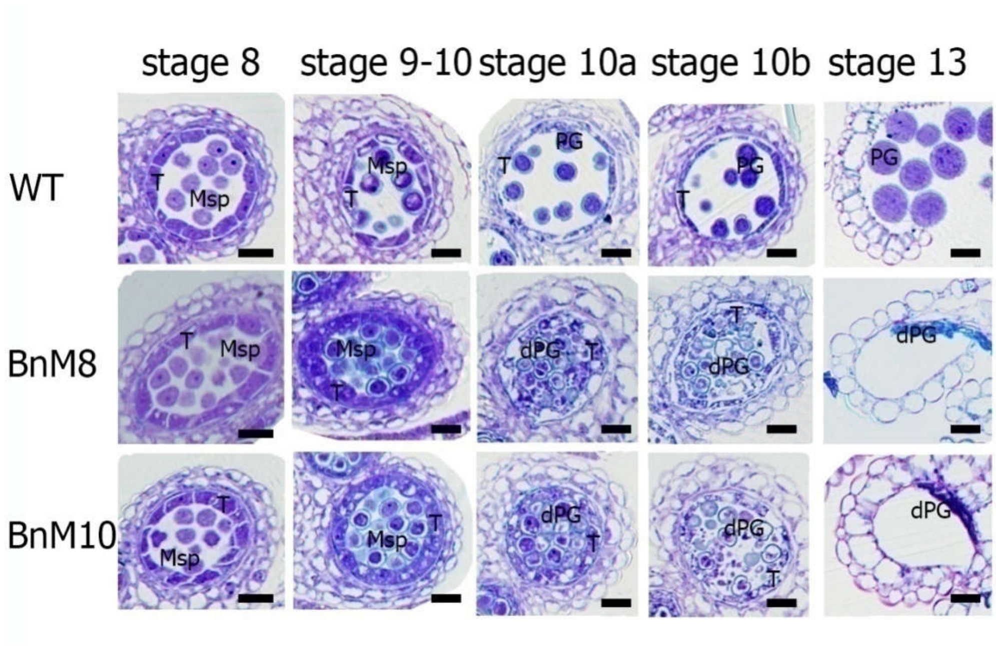

Figure S2. Semi-thin sections of anthers from the wild type, the *BnA9::BnaC.MAGL8.a* plants and the *BnA9::BnaA.MAGL10.a* plants. Anthers from the *BnA9::BnaC.MAGL8.a* plants and the *BnA9::BnaA.MAGL10.a* plants showed similar development progresses. In the transgenic plants, the tapetal cells accumulated small vacuoles at stage 9 and the tapetum vacuolation became severe in later stages. Tapetal cells and microspores degenerated in stage 10 and the degenerated pollen grains adhered to locule wall at stage 13. BnM8, *BnA9::BnaC.MAGL8.a*; BnM10, *BnA9::BnaA.MAGL10.a*; dPG, degenerated pollen grains; Msp, microspores; PG, pollen grain; stage 10a, middle stage 10; stage 10b, late stage 10; T, tapetum; WT, wild type. Bars = 12 μm.

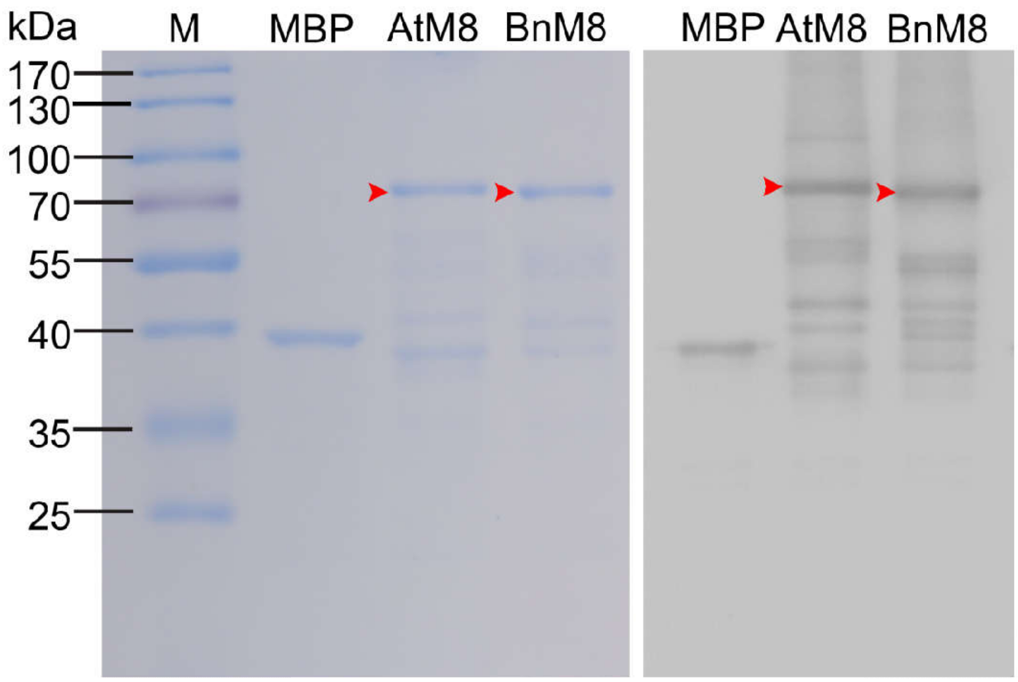

Figure S3. Recombinant protein purification. The purified proteins were separated on a 8% SDS-PAGE gel. Then the proteins were detected with Coomassie blue staining (the left panel) and western blot (the right panel). The recombinant proteins are indicated with red arrowheads. M, molecular weight standards; MBP, maltose binding protein; AtM8, MBP:AtMAGL8 recombinant protein; BnM8, MBP:BnC.MAGL8.a recombinant protein.

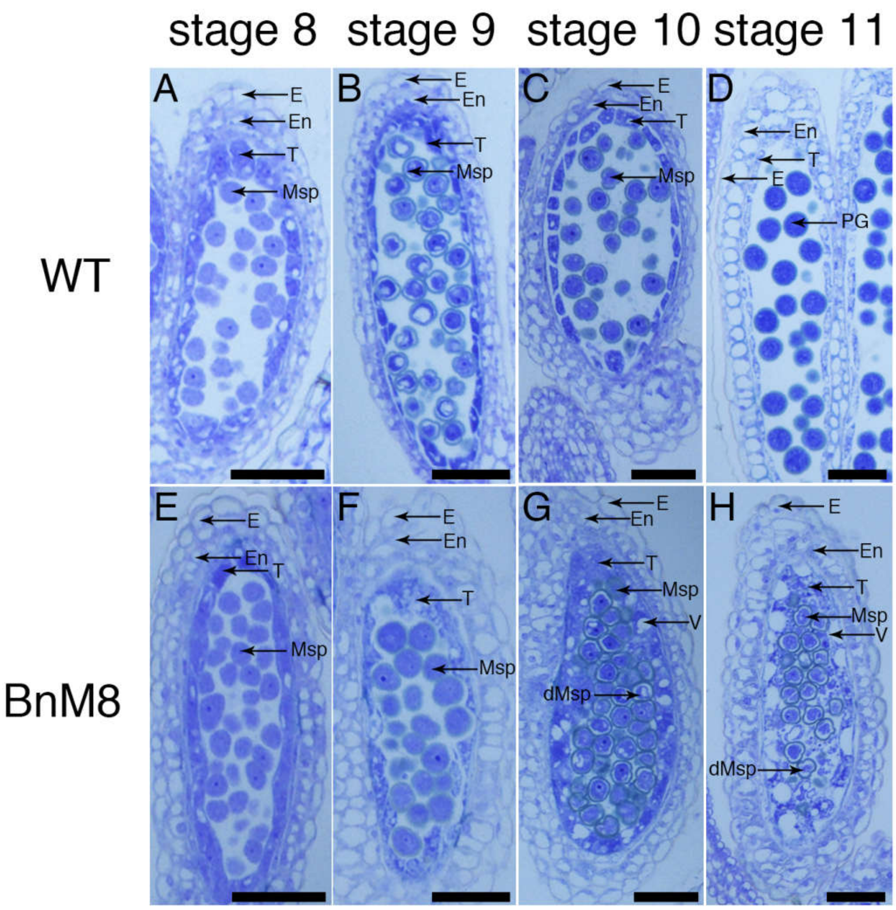

Figure S4. Longitudinal semi-thin sections of anthers from the wild type and *BnA9::BnaC.MAGL8.a* plants. (A) to **(D)**: semi-thin sections from the wild type at pollen development stage 8 **(A)**, stage 9 **(B)**, stage 10 **(C)** and stage 11 **(D)**. **(E)** to **(F)**: semi-thin section from the *BnA9::BnaC.MAGL8.a* plants at pollen development stage 8 **(E)**, stage 9 **(F)**, stage 10 **(G)** and stage 11 **(H)**. BnM8, *BnA9::BnaC.MAGL8.a*; dMsp, degenerated microspores; E, epidermis; En, endothecium; Msp, microspore; PG, pollen grain; T, tapetum; V, vacuole; WT, the wild type. Bars = 40 μm.

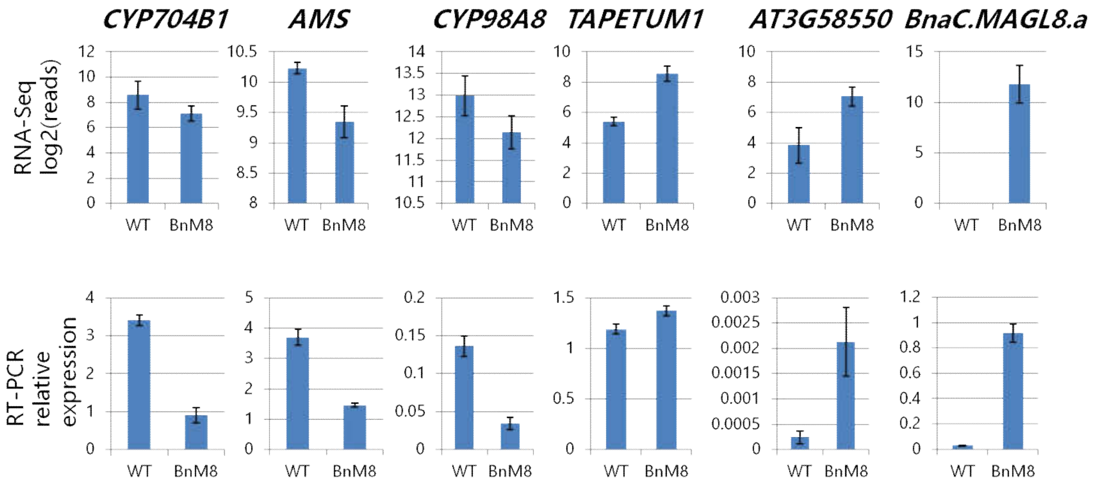

Figure S5. Validation of six differentially expressed genes by real-time PCR. The upper panel shows the results from transcriptom analysis (RNA Sequencing, RNA-Seq) result. The values are log2 transformed reads for respective genes and the standard errors were calculated from three biological replicates. The lower panel shows the results from real-time PCR (RT-PCR) analysis. The values are relative expression value for respective genes and the standard errors were calculated from three biological replicates.

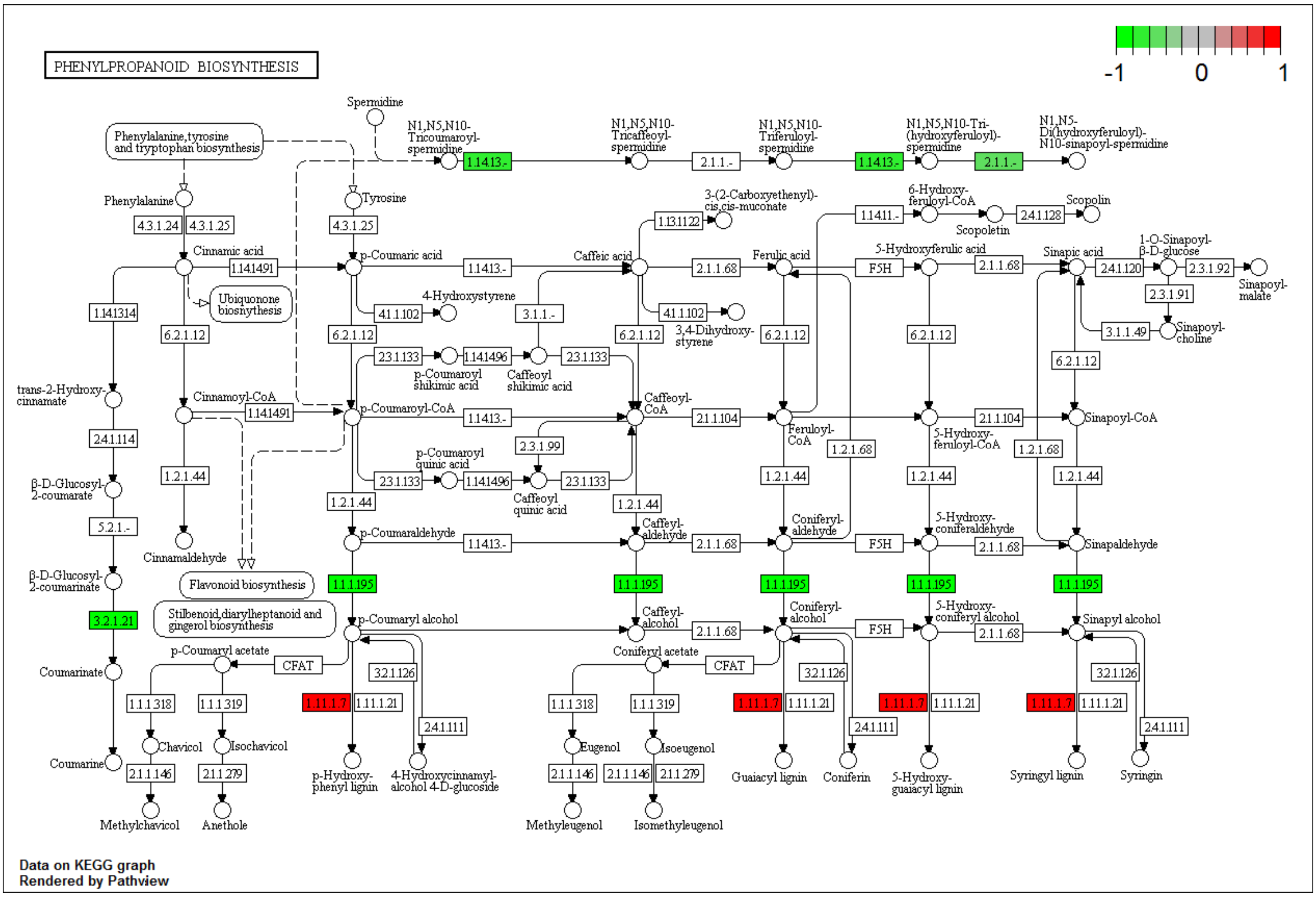

Figure S6. Differentially expressed genes in phenylpropanoid biosynthesis pathway. Green boxes represent down-regulated genes and red boxes represent up-regulated genes. The boxes were colored by the log2FoldChange value of correlated genes. The gene expression data for this figure is presented in Table S6. KEGG pathway visualization was performed with Pathview (Luo and Brouwer, 2013)
